# Seed Treatment Alternatives with Improved Ecological Profiles for Controlling Wireworms in Maize and Early-Season Sugar Beet Pests

**DOI:** 10.3390/plants15101488

**Published:** 2026-05-13

**Authors:** Renata Bažok, Darija Lemić, Dragan Bubalo, Ante Kasap, Milorad Vojvodić

**Affiliations:** 1 Division of Plant Medicine, University of Zagreb Faculty of Agriculture, Svetošimunska Cesta 25, 10000 Zagreb, Croatia; dlemic@agr.hr; 2 Division of Animal Sciences, University of Zagreb Faculty of Agriculture, Svetošimunska Cesta 25, 10000 Zagreb, Croatia; dbubalo@agr.hr (D.B.); akasap@agr.hr (A.K.); 3 INOVINE d.d., 10000 Zagreb, Croatia

**Keywords:** maize, seed treatment, sugar beet, sustainable pest control

## Abstract

The ban on neonicotinoid seed treatments in the EU has created major challenges for maize and sugar beet production, as these chemicals have been highly effective in controlling early-season pests, including wireworms (*Agriotes* spp.), sugar beet weevil (*Asproparthenis punctiventris* Germar) (SBW) and sugar beet flea beetles (*Chaetocnema tibialis* Illiger) (SBFB). However, adequate alternatives have not yet been introduced. The aim of this research was to get insights on the biological activity of insecticides with distinct modes of action and comparatively more favorable ecotoxicological profiles than neonicotinoids, chlorantraniliprole, spinosad, and azadirachtin, applied as seed treatments in maize and sugar beet against wireworms in maize and against SBW and SBFB in sugar beet. In laboratory trials, each insecticide was tested as a seed treatment at three different doses. Thiamethoxam was included as the standard treatment (positive control). Among the tested insecticides, spinosad seed treatment showed the highest efficacy against wireworms and was superior to both the standard insecticide and chlorantraniliprole, while azadirachtin showed no effect. None of the tested insecticides provide satisfactory control of SBW. In contrast, SBFB responded to all three insecticide treatments, with efficacy comparable to, or even better than, the standard insecticide. These results suggest that chlorantraniliprole, azadirachtin, and spinosad may all represent promising candidates for sugar beet seed treatment to protect young plants against SBFB. Future research should focus on developing seed treatment formulations and field and semi-field trials as well as evaluating combinations of active ingredients and their suitability for integration into IPM programs. These findings provide a basis for further development of seed treatment strategies aimed at reducing dependence on neonicotinoids in maize and sugar beet production.

## 1. Introduction

Treating seeds with insecticides to protect plants against soil pests offers several advantages, but also some disadvantages. The main advantage of this method is the reduced amount of active substance used on a much smaller area compared to applying it on the entire field [1], which mitigates the negative impact on the environment. Using a less active substance results in lower residue levels and a reduced risk of leaching into groundwater [1,2]. Treating a smaller area also means less exposure and a reduced negative effect on beneficial soil organisms such as springtails (Collembola), ground beetles (order Coleoptera, family Carabidae), and rove beetles (order Coleoptera, family Staphylinidae). Additionally, the lower amount of active substance per hectare leads to reduced costs per unit area. Since the seed is treated at the seed company, the risk of possible toxic effects for farmers handling the treated seed is also reduced [3].

However, there are several disadvantages of seed treatments [1,3]. These include possible errors during the seed treatment and sowing processes that can reduce effectiveness or cause negative environmental effects, the need for careful selection of insecticides suitable for this method, and the preventive and often indiscriminate sowing of treated seeds without monitoring or a documented assessment to justify their use.

Seed treatment with insecticides as an effective method of protecting against soil pests began in the mid-1950s, when the first seed treatment formulations based on chlorinated hydrocarbons—aldrin, dieldrin, heptachlor, and lindane—were developed [4].

The era of development and widespread use of insecticide-treated seeds began with the introduction of active ingredients from the neonicotinoid group—imidacloprid, clothianidin, and thiamethoxam—in the mid-1990s. In addition to their use in seed treatment, neonicotinoids are also foliar applied for control of many pests. The three active ingredients approved for seed treatment exhibit strong systemic action and, besides their effect on wireworms, are highly effective against early-season pests such as aphids and flea beetles. As a result, seed treatment with neonicotinoids has become standard practice in certain crops, including sugar beet and maize. In the United States, neonicotinoid-treated seeds became the standard in major row crops. By 2011, approximately 79–100% of maize and 34–44% of soybean hectares were planted with insecticide-treated seed [5]. During the same period, the proportion of fields sown with treated seeds in the European Union for maize, oilseed rape, and other field crops averaged around 30%, while for sugar beet it was nearly 100% [6]. However, many authors [7,8,9,10] have reported that there is no clear evidence confirming a significant reduction in wireworm damage or yield preservation in infested plots sown with neonicotinoid-treated seeds. Despite the growing interest in reduced-risk and biologically derived insecticides, information on their suitability for seed treatment in arable crops remains limited, particularly when efficacy must be ensured simultaneously against both soil pests and early-season foliar pests. This knowledge gap is especially relevant for sugar beet, where successful crop establishment depends on early protection against multiple pest groups.

Based on three studies organized by EFSA [11,12,13] that reviewed all relevant research on neonicotinoids worldwide, on 27 April 2018, the European Commission permanently banned the use of these three active substances from the neonicotinoid group for all uses, including seed treatment, except for permanent greenhouse crops.

The ban on neonicotinoids for seed treatment led to increased attacks and frequent problems in the production of crops affected by numerous foliar pests, such as oilseed rape and sugar beet, resulting in decreased yields and, consequently, a reduction in the area sown with these crops [14,15]. For this reason, EU member countries (e.g., France [16], Slovakia [17], Romania [18], Poland [19] and others) issued emergency authorization for the treatment of sugar beet seeds with neonicotinoids until 2022. In 2020, the European Commission asked EFSA to assess whether the emergency authorizations granted by the Member States were justified because there was a danger to crops “which cannot be contained by any other reasonable means,” in line with the EU Plant Protection Products Regulation. EFSA concluded that in all 17 cases, the emergency authorizations were justified, either because no alternative products or methods—chemical or non-chemical—were available or because there was a risk that the pest could become resistant to available alternative products [20]. However, by its judgment of 19 January 2023, the European Court of Justice (CJEU) [21,22] ruled that Member States cannot issue emergency authorizations for the treatment of seeds with prohibited neonicotinoids, as the placing of seeds on the market and use of seeds treated with neonicotinoids is strongly prohibited by EU regulations. Therefore, the previously issued emergency authorizations were deemed contrary to European law, and their legal justification was annulled by the court. Following the judgment, no further emergency authorizations for the treatment of seeds with neonicotinoids are issued in the EU. The Court [21,22] warned Member States that they must use the alternative pest control methods currently available and should work to develop improved alternative methods.

This judgment particularly threatens sugar beet production, which, in addition to soil pests such as wireworms, is attacked in the early stages of development by numerous pests of aboveground organs that feed on the leaf surface (sugar beet flea beetle, *Chaetocnema tibialis* Illiger, SBFB, and sugar beet weevil *Asproparthenis punctiventris* Germar 1824, SBW) or transmit viral diseases (aphids) [23]. These pests have been successfully controlled by sowing seeds treated with neonicotinoids. Alternative methods of protection against these pests include foliar application of insecticides from the pyrethroid group, which have a strong negative effect on non-target organisms, primarily natural enemies, and whose frequent use leads to the development of resistance. The success of controlling these pests by foliar application of insecticides also largely depends on timely treatment, which can be compromised by adverse weather conditions and is often limited by the small leaf area available for application. Therefore, it is necessary to find alternative methods of protection against these pests or to identify alternative active substances with acceptable ecotoxicological properties that can be used as seed treatments and are effective against wireworms as well as pests of the aboveground organs of sugar beet, such as beet weevil and flea beetles.

Among the existing active substances with potential for use as seed treatments, due to their systemic action, are chlorantraniliprole and cyantraniliprole. Both substances belong to the group of ryanodine receptor modulators (group 28 according to the IRAC classification), specifically the group of compounds known as diamides [24]. Due to its systemic translocation, low potential for bioconcentration, and low toxicity, chlorantraniliprole is selective for beneficial arthropods and is considered suitable for use in integrated pest management. However, its moderate toxicity to fish and high toxicity to aquatic invertebrates are negative aspects of this substance [24]. Data from previous studies show that chlorantraniliprole applied as a seed treatment does not provide satisfactory efficacy against wireworms [25,26]. According to some results, this substance shows some effect on pests of aboveground organs of rice, such as *Lissorhotrus oryzaephilus* (Kuschel) [27,28,29], *Scirpophaga incertulas* (Walker) [30], *Eoreuma loftini* (Dyar) [29], and *Spodoptera frugiperda* (J. E. Smith) and *Diatraea saccharalis* F. [28]. No products containing chlorantraniliprole for seed treatment are registered in the EU, while such products exist in other parts of the world, such as the USA and Canada. The insecticides Lumia and Dermacor X-100, both based on chlorantraniliprole, are registered in Canada and the USA by Corteva, respectively [31].

Spinosad is a fermentation product of the soil bacterium *Saccharopolyspora spinosa* Mertz & Yao, 1990, with its derivatives spinosyn A (85%) and spinosyn D (15%) [32]. It belongs to the group of biological insecticides known as naturalites, which act on pests by contact and ingestion [24]. Spinosad is less toxic to beneficial organisms than other insecticides [33,34] and has low toxicity to mammals and the environment [35]. Toxicity of spinosad to honeybees can be high, and if honeybees are directly exposed to concentrations recommended for foliar application under field conditions, mortality can reach 100% [36]. However, Mayes et al. [37] demonstrated that once spinosad residues have dried on plant foliage, generally 3 h or less, the risk of spinosad to honeybees is negligible. Spinosad is used in agricultural pest control and also has sanitary applications, showing effectiveness against many pests [34] that feed on leaves, such as lepidoptera, leaf miners, and thrips [38]. It is also very effective in controlling the cherry fruit fly *Rhagoletis indifferens* Curran by foliar application [35]. As a seed treatment insecticide, spinosad is used only for onion seed to control the onion fly *Delia antiqua* Meigen, where very good results have been achieved [39,40]. Good results in controlling wireworms (*Agriotes lineatus* L. and *Agriotes obscurus* L.) under laboratory conditions were reported by Ericsson et al. [41]. However, research by Van Herk et al. [42] in laboratory conditions showed poor efficacy of spinosad on *Agriotes obscurus* larvae. Van Herk et al. [25] reported that treatment of wheat seeds with spinosad in laboratory conditions led to temporary morbidity of wireworm larvae, which later recovered. Cleveland et al. [33] consider that the negative effects on honeybee colonies exposed to typical environmental concentrations of this insecticide are minimal. Negative effects on bumblebees are also considered minimal [43]. Application of this insecticide as a seed treatment further minimizes possible negative effects on pollinators. Granulated formulation of spinosad (4 g/kg) has been registered for wireworm control in maize and potato in Croatia since 2022 [44].

Azadirachtin is a botanical insecticide derived from the neem plant, *Azadirachta indica* Juss, which has been used for thousands of years in medicine, cosmetics, and pest control, both outdoors and indoors [45]. Neem oil contains more than 100 biologically active compounds, with limonoids being the most abundant; among these, azadirachtin is the most significant [46]. The use of azadirachtin reduces pest populations by negatively affecting their feeding, development, and reproduction [47]. It is effective in suppressing populations of shield moths, butterfly caterpillars, thrips, and a total of 200 types of insects, mites, and nematodes [48]. According to Kadoić Balaško et al. [49], azadirachtin was efficient against Colorado potato beetle (*Leptinotarsa decemlineata* Say) larval stages, causing high mortality and significantly reducing damage to potato leaves, but it was less effective against adults. For western flower thrips adults, azadirachtin was not highly effective, causing only 50% mortality after three days. Higher doses of azadirachtin also reduced oviposition, which can prevent further population growth. Good effectiveness of azadirachtin used in seed treatment was found in controlling thrips and caterpillars on cotton [50]. As a botanical insecticide with a more favorable ecotoxicological profile, azadirachtin is a promising candidate that, if efficacy is satisfactory, could replace previously approved insecticides for seed treatment.

Maize and sugar beet were included as representative field crops in which seed treatment has historically played an important role in early pest management. While wireworms were studied in maize as a major soil pest target, sugar beet was used to assess the activity of the same seed treatment candidates against key early-season aboveground pests. This combined approach allowed the comparative evaluation of alternative active substances across different pest guilds relevant to seed treatment-based crop protection.

This study aimed to get insights on biological activity and determine the effectiveness of insecticides with a more favorable ecotoxicological profile—chlorantraniliprole, spinosad, and azadirachtin—applied by treating maize and sugar beet seeds against wireworms in maize and against the two most important pests that attack sugar beet in the early stages of development: the sugar beet flea beetle (*Chaetocnema tibialis*) (SBFB) and the beet weevil (*Asproparthenis punctiventris*) (SBW). It was hypothesized that insecticides with more favorable ecotoxicological characteristics could provide selective but useful protection against some of the tested pest species, although their efficacy would likely differ depending on pest biology, feeding behavior, and exposure pathway.

## 2. Results

### 2.1. Wireworms on Maize

In laboratory experiments, an average of 4.4 wireworms survived in the untreated control group in 2016 and 4.5 wireworms in 2017. All wireworms survived for the entire duration of the experiment when exposed to the insecticide azadirachtin. For this reason, the data for azadirachtin was excluded from statistical analysis. After conducting an analysis of variance and factorial analysis, it was determined that the efficacy of insecticides on wireworms depends on the active substance (*p* < 0.0001, HSD = 6.45), dose (*p* < 0.0001, HSD = 5.07), and their interaction (*p* < 0.0001, HSD = 14.52) (Table 1 and Table 2).

The standard insecticide thiamethoxam, applied at all three doses in 2016, resulted in an average efficacy of 43%, while spinosad had an average efficacy of 60%. In 2017, the efficacy of insecticides was found to depend on the active substance (*p* < 0.0001, HSD = 1.31) but not on the dose (*p* > 0.01). Additionally, the interaction between dose and active substance significantly affected efficacy (*p* < 0.0001, HSD = 6.02) (Table 1).

In 2017, the efficacy of all insecticides was lower compared to 2016, particularly for the standard insecticide thiamethoxam and the alternative insecticide chlorantraniliprole. Thiamethoxam achieved a lower average efficacy of 16% in 2017 compared to 43% in 2016. The efficacy of spinosad was slightly lower than in 2016. Chlorantraniliprole showed extremely low efficacy, similar to the results from 2016, while azadirachtin, which was not included in the 2016 experiments, did not achieve any efficacy and was at the level of the untreated control. Although these were laboratory experiments, the influence of external factors, most notably the developmental stage of the larvae collected for the experiments in the two years of the study, could not be avoided. Nevertheless, across both experimental years, spinosad consistently performed better than the other tested alternatives, indicating a more stable toxic effect on wireworms.

### 2.2. Sugar Beet Pests

#### 2.2.1. Sugar Beet Weevil

The results of the efficacy of insecticides against sugar beet weevil are shown in Table 3.

**Figure 1:**
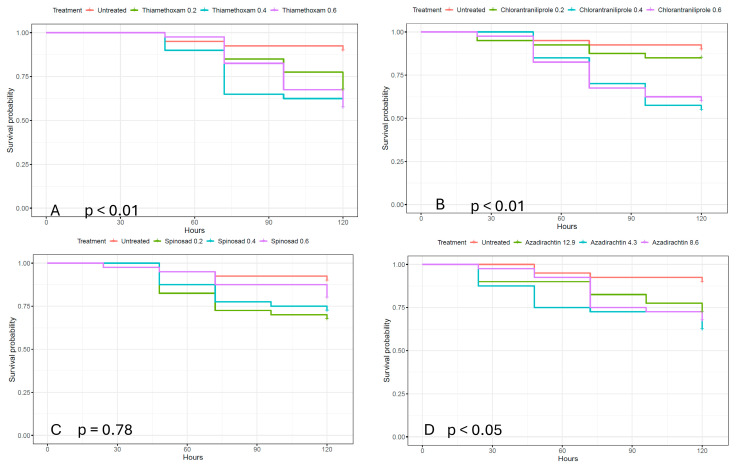
Survival rates of sugar beet weevils fed with plants treated with thiamethoxam (**A**), chlorantraniliprole (**B**), spinosad (**C**), and azadirachtin (**D**), and the results of tests for differences in survival rates among groups, as determined by the global “log-rank” test, are shown as *p*-values below each figure.

Survival rates of sugar beet weevils (SBW), fed on sugar beet grown from seed treated with different insecticides, are shown by Kaplan–Meier curves in Figure 1.

The results of the “pair-wise” test and comparison of the probability of survival (*p*) for sugar beet weevils fed on plants treated with different insecticides 120 h after experimental setup are shown in Table 4.

The survival rates of SBW fed on sugar beet grown from seeds treated with the active substances thiamethoxam, chlorantraniliprole, spinosad, and azadirachtin, each at three different doses and shown by Kaplan–Meier curves in Figure 1, indicate the first mortality in all observed weevils’ groups for all active substances within 24 h. The figure also shows higher mortality in all treated weevils’ groups (exposed to all applied insecticides) compared to the untreated group throughout the observation period.

Testing differences in mortality dynamics with global log-rank tests showed significant differences between the survival probabilities of weevils’ groups treated with thiamethoxam (*p* < 0.01), chlorantraniliprole (*p* < 0.01), and azadirachtin (*p* ≤ 0.05) but not for spinosad (*p* = 0.078).

Subsequent multiple individual log-rank tests (Table 4) revealed significant differences in mortality between the untreated group and the groups treated with thiamethoxam at all doses, chlorantraniliprole at the medium and higher doses, and azadirachtin at lower and medium doses 120 h after exposure. For the lower dose of chlorantraniliprole, all doses of spinosad, and the higher dose of azadirachtin, the differences were not significant. For chlorantraniliprole, significant differences in survival probability were found between the higher and medium doses compared to the lower dose (*p* ≤ 0.05 for the medium and *p* < 0.05 for the higher dose). Differences between the medium and higher doses of chlorantraniliprole, as well as differences between different doses of all other insecticides used in the experiment, were not significant (Table 3).

However, the survival probability of SBW after feeding on plants treated with all tested insecticides is over 0.5, which is too high to conclude that the tested insecticides would ensure satisfactory control of SBW.

#### 2.2.2. Sugar Beet Flea Beetle

The results of the efficacy of insecticides against the sugar beet flea beetle are shown in Table 5.

**Figure 2:**
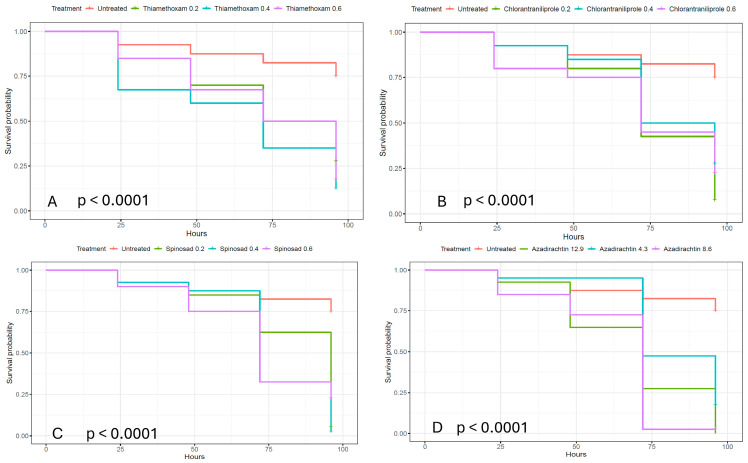
Survival rates of sugar beet flea beetles (SBFB) fed with plants treated with thiamethoxam (**A**), chlorantraniliprole (**B**), spinosad (**C**), and azadirachtin (**D**), and the results of tests for differences in survival rates among groups, as determined by the global “log-rank” test, are shown as *p*-values below each figure.

Survival rates of sugar beet flea beetles (SBFB) fed on sugar beet grown from seed treated with different insecticides are shown by Kaplan–Meier curves in Figure 2.

The results of the “pair-wise” test and comparison of the probability of survival (*p*) for sugar beet flea (SBFB) beetles fed on plants treated with different insecticides 96 h after experimental set up are shown in Table 6.

The survival rates of SBFB fed on sugar beet grown from seeds treated with the active substances thiamethoxam, chlorantraniliprole, spinosad, and azadirachtin, each at three different doses and shown by Kaplan–Meier curves in Figure 2, indicate the first mortality in all observed flea beetle groups for all active substances within 24 h. The figure also shows higher mortality in all treated flea beetle groups (exposed to all applied insecticides) compared to the untreated group throughout the observation period.

Testing differences in mortality dynamics with global log-rank tests showed significant differences between the survival probabilities of SBFB groups treated with all four applied insecticides (*p* < 0.0001).

Subsequent multiple individual log-rank tests (Table 4) revealed significant differences in mortality between the untreated group and the groups treated with all insecticides at all doses. Only for azadirachtin, significant differences in survival probability were found between the higher and medium doses compared to the lower dose (*p* < 0.01). Differences between the higher and medium doses of azadirachtin, as well as differences between different doses of all other insecticides used in the experiment, were not significant (Table 5).

In contrast to SBW, SBFB showed consistently high susceptibility to all tested insecticides, suggesting that this pest is a more promising target for alternative seed treatment strategies.

## 3. Discussion

The number of registered active insecticide substances in Europe is constantly decreasing. Between 1987 and 2018, the number of insecticide active substances in Croatia decreased by 40%, and the number of formulated products by 50% [51]. Bažok [52] reports that in 2013, seven active substances were approved for seed treatment, with a total of 13 formulated products on the market, while in 2026, only three active substances and four products [44] were approved. To ensure effective protection of sugar beet and other crops from pests attacking aboveground organs during early growth, previously controlled with systemic insecticides from the neonicotinoid group, which are now banned, foliar insecticides from the pyrethroid group are now used. This has led to increased resistance development in a larger number of pests [14]. Therefore, there is a need to find environmentally friendly insecticides with different mechanisms of action that can effectively control both soil pests and early-season pests. This research included wireworms, which attack many crops, as well as the sugar beet weevil (SBW) and sugar beet flea beetle (SBFB), important early-season pests of sugar beets. Chlorantraniliprole, spinosad, and azadirachtin were investigated as alternative insecticides, with thiamethoxam from the neonicotinoid group used as the standard.

The efficacy levels observed here should be considered indicative of biological potential rather than direct predictors of field performance of applied insecticides. One reason is that the insecticides used in our investigations were not formulated as seed treatment formulations (FS or WS); they were formulated as foliar treatment insecticides (WG and SC), and the experimental seed dressing included an auxiliary substance to ensure seed coating. The fact that the applied formulations lack the polymers and adhesive agents necessary to ensure stable adherence to seeds could jeopardize the efficacy of the insecticides [53]. Our intention was not to evaluate the efficacy of formulated products, but rather to obtain indicative information on the possible biological activity of the applied insecticides and to select candidates for further investigation. For such studies, further development of seed treatment formulations would be needed, and research should be implemented in field and semi-field studies as they can confirm whether the observed effects can be translated into reliable crop protection under practical production conditions.

This study found considerable variability in the efficacy of all applied insecticides achieved against wireworms across different years. One possible reason for this variability is the relatively small number of replicates and larvae used in the experiments. Although the experiment primarily used larvae of the species *A. ustulatus*, a proportion of the larvae (between 10% and 20%) belonged to other species that may express different sensitivities. These factors could have influenced both the efficacy of the insecticides against wireworms and the observed variability. This also highlights the preliminary nature of these results and the need for further research.

In the research conducted, thiamethoxam did show low to moderate efficacy against wireworms. Numerous authors have reported the same issue [7,10,54,55,56,57]. Most of these authors point out that sowing seeds treated with neonicotinoids does not reduce pest occurrence or increase the profitability of plant production. Moreover, Labrie et al. [8] state that the benefit from sowing seeds treated with neonicotinoids in protecting field crops is less than 5%, while Smith et al. [9] report that under conditions of severe wireworm infestation in soybean and maize crops, sowing seeds treated with neonicotinoids preserved maize yields by 8% and soybean yields by 6%.

In treatments where thiamethoxam was applied, significantly higher mortality of SBW and SBFB was observed compared to the untreated control (*p* < 0.05 for all three doses). No differences were found between the different applied doses. The effectiveness of thiamethoxam is comparable to the results reported by Virić Gašparić et al. [23] using a combined treatment of sugar beet seeds with thiamethoxam and tefluthrin under field conditions.

Chlorantraniliprole, an insecticide from the anthranilic diamide group, applied at doses of 2, 3.5, and 5 g per 1 kg of seed, did not show satisfactory results in controlling wireworms (Table 2), which is consistent with the findings of van Herk et al. [42] on wheat seed treatment.

The effectiveness of the same insecticide on early-growth pests of sugar beet varied depending on the pest. Although significantly higher mortality of SBW was observed after feeding on plants treated with medium and high doses (*p* < 0.01), this was not sufficient for effective protection against this pest under field conditions as the efficacy was around 30% (Table 3). All applied doses of chlorantraniliprole significantly increased SBFB mortality compared to the untreated control, and the differences between doses were not significant. All chlorantraniliprole treatments achieved a similar effect (about 80%), which is comparable to the standard insecticide thiamethoxam and can be considered acceptable under field conditions (Table 5). The results show that the effectiveness of chlorantraniliprole applied as a seed treatment depends significantly on the insect species being controlled. In this case, important morphological and physiological characteristics of the insect are determined by systematic category, body size and structure, and feeding behavior. Both insects studied are beetles (Coleoptera) that feed in a similar way, with the main difference being feeding intensity and body size. SBW has larger body dimensions and greater feeding capacity compared to SBFB, so the better effectiveness on SBFB can be attributed to these differences. The good effectiveness of chlorantraniliprole applied as a seed treatment on Coleoptera pests was demonstrated by Villegas et al. [28], who found satisfactory control of the rice pest *Lissorhoptrus oryzophilus* Kuschel, while Lanka et al. [27] found that feeding on plants grown from treated seeds led to reduced oviposition of the same species. Rice seed treatment with chlorantraniliprole also provided effective control against Lepidoptera pests *Spodoptera frugiperda* J.E. Smith and *Diatraea saccharalis* F. [28], as well as *Scirpophaga incertulas* Walker and *Cnaphalocrocis medinalis* Guenee [30]. Chlorantraniliprole applied as a seed treatment to maize was also effective against the *Agrotis ypsilon* L. [58] and *Spodoptera frugiperda* [59,60].

Chlorantraniliprole is approved for foliar application in the European Union [61] and for seed treatment in some non-EU markets [10]. It is permitted for treating maize seeds in certain states of the USA [31], as well as in Ontario and Quebec, Canada [62]. Four formulated products based on chlorantraniliprole have been registered in the Republic of Croatia; however, none, as in other EU Member States, have a permit for seed treatment.

Due to its systemic nature, low toxicity, low potential for bioconcentration, and selectivity for beneficial arthropods, chlorantraniliprole is considered suitable for use in integrated plant protection and could be a good substitute for neonicotinoids. However, as these results and those of other authors show, it is effective only against a limited number of pests, which should be further investigated. Given that chlorantraniliprole could contaminate pollen and nectar due to its systemic nature, potentially posing a risk to beneficial species, it is necessary to conduct a long-term assessment of its impact on beneficial arthropods [63].

Research has shown that spinosad applied as a seed treatment is effective against wireworm larvae, achieving about 70% efficacy, which is higher than the standard insecticide, thiamethoxam. Significant evidence for the efficacy of spinosad as a soil treatment was found in field trials conducted in Northeast Italy [64]. They demonstrated that spinosad applied in-row during maize planting effectively prevented damage by *Agriotes* spp. larvae and protected overall crop yield. The granular spinosad formulation, applied in furrows at onion planting, significantly increased plant density 20 days after treatment [65]. At the same time, its liquid formulation, applied as a soil treatment during planting, did not provide such good results [65]. Good results in laboratory conditions were obtained by Ericsson et al. [41] by mixing spinosad at sublethal doses with the entomopathogenic fungus *Metharhizium anisopliae* (Metschn.) Sorokīn in controlling wireworms of the species *Agriotes lineatus* L. and *Agriotes obscurus* L. In contrast, research conducted by Van Herk et al. [25] in laboratory conditions showed low efficacy of spinosad against wireworms of the species *A. obscurus*. Several studies reported that spinosad is largely ineffective when used as a solitary seed treatment for crops like wheat and maize [42], as in laboratory contact bioassays, wireworms exposed to spinosad-treated wheat seeds often became moribund (intoxicated) within 24 h but did not suffer high levels of sustained mortality. This indicates that while spinosad can temporarily reduce the pests’ attack, it does not reliably reduce the overall wireworm population in the field [42]. The granular formulation of spinosad is approved in Croatia for use as a soil insecticide in maize for wireworm control. Given the differences between our results and those of other authors regarding established efficacy on wireworms, additional research would be useful.

Treatments of sugar beet seeds with spinosad did not significantly affect SBW mortality, regardless of the applied dose (*p* > 0.05, Table 4). Bažok et al. [66] reported satisfactory effectiveness of spinosad on SBW after foliar application in the laboratory. Since their research used spinosad as a foliar treatment and the current study used it as a seed treatment, differences are expected.

In contrast to SBW, spinosad seed treatments significantly affected SBFB mortality compared to the untreated control. Although statistically significant differences in mortality at different doses were not determined, it is evident that spinosad treatments at doses of 0.2 and 0.4 mg d.t./seed had the greatest impact (*p* < 0.01), with SBFB mortality above 95% (Table 5), which is significantly higher than the standard insecticide. The recent literature contains no studies on the effect of spinosad on SBFB. Seed treatment with spinosad is mainly used on onions, where good results have been achieved in controlling the onion fly (*Delia antiqua* Meigen) [39].

The results of this research show the good potential of spinosad applied as a seed treatment for protecting sugar beet from SBFB. Further studies confirming the effectiveness of spinosad on pests of aboveground organs in other field crops whose seeds were treated with neonicotinoids before their ban (e.g., sunflower, oilseed rape) would enable an adequate assessment of whether spinosad, although perhaps of limited effectiveness, is a good alternative for seed treatment.

Azadirachtin, applied as a seed treatment in our research, was not effective against wireworms. Since even granular formulations of azadirachtin do not show good efficacy on wireworms, because azadirachtin-treated soil has been shown to be repellent to wireworm species such as *Melanotus communis* (Gyllenhal, 1817) [67], this result is somewhat expected. To address the problem of azadirachtin repellency, Humbert et al. [68] investigated the co-encapsulation of *Saccharomyces cerevisiae* Meyen ex E.C. Hansen and neem extract and showed that this is a promising approach for developing attract-and-kill formulations for wireworm control. Azadirachtin effectively suppresses larvae of the western corn rootworm (*Diabrotica virgifera virgifera* LeConte) [69,70].

Although seed treatments with azadirachtin had a significant effect on SBW mortality compared to the untreated control (*p* < 0.05), the effect of the applied doses was extremely low (between 15 and 30%) and cannot be considered satisfactory for practical use (Table 3).

Contrary to the established weak effect of azadirachtin on SBW, its effect on SBFB was satisfactory (*p* < 0.01). All three applied doses significantly reduced survival compared to the untreated control. The high and medium doses of azadirachtin resulted in lower survival than the low dose, which also achieved an effect comparable to the standard insecticide (Table 5). No scientific papers were found in the available literature on the effectiveness of azadirachtin applied by seed treatment on SBW and SBFB, but good efficacy of this insecticide, when used as a seed treatment, has been established for controlling thrips and caterpillars on cotton [50].

Considering previous research and our results, we can confirm that there is potential for the development of neem-based botanical soil insecticides not only for arable crops such as maize, as suggested by Toepfer et al. [69], but also for other crops. These products might become, if more soil insecticides are phased out, a promising and safer solution as part of the integrated pest management toolkit against soil insects [69]. Additionally, granular formulations of neem and seed treatments are effective against nematodes such as *Meloidogyne incognita* Kofoid et White (Chitw.) [71], *Meloidogyne javanica* (Treub) Chitw. [72], and others, including various arthropods such as termites and mealy bugs [73]. Therefore, it is very likely that new formulations of azadirachtin intended for soil or seed treatment can be expected in the future. According to Daly [74], an extremely rapid breakdown could be a problem and the reason why azadirachtin might not be appropriate for application to the soil as a commercial insecticide. To solve this problem, the granules for a controlled release of azadirachtin were developed [74]. Azadirachtin was shown to be sufficiently mobile within soil and plants for movement from a granule source to the leaf tissue [74], and it could be expected that the effects of such formulations will target not only harmful soil species but also aboveground pests, including SBFB.

From an IPM perspective, the value of these alternative seed treatments does not depend solely on their direct insecticidal effect, but also on their compatibility with other control tactics, including pest monitoring, threshold-based foliar interventions, conservation of natural enemies, and resistance management strategies. The results indicate that no single tested alternative can presently replace neonicotinoid seed treatments across all relevant pests. However, some of the tested substances, particularly spinosad for wireworms and all three alternatives for SBFB, may represent useful components of a more diversified early-season pest management strategy.

## 4. Materials and Methods

### 4.1. Wireworms on Maize

Research was conducted in 2016 and 2017 under laboratory conditions. The following insecticide products were used in the experiment: (i) Coragen 20 SC (Corteva Agrisciences, Indianapolis, IN, USA), based on chlorantraniliprole from the anthranilic diamide group; (ii) Laser 240 KS (CortevaAgrisciences, Indianapolis, IN, USA), based on spinosad from the spinosyn group; and (iii) NeemAzal (Trifolio-M GmbH, Lahnau, Germany), based on azadirachtin. The insecticide Actara 25 WG (Syngenta, Basel, Switzerland), based on thiamethoxam from the neonicotinoid group, was used as a standard. An untreated control was also included. In 2016, the experiment included 10 treatments: three insecticides at three different doses and an untreated control. The mean applied dose of all insecticides matched the permitted doses. Higher and lower doses of thiamethoxam, chlorantraniliprole, and spinosad were increased and decreased, respectively, by 43%. For azadirachtin, the average dose was increased or decreased by 50%. In 2017, the experiment included 13 treatments: four insecticides at three different doses and an untreated control. The list of insecticide products, doses, and active ingredients is shown in Table 7.

For maize seed treatment, a suspension of water and Agrocoat-TH Intensivrot 3855 in a 55:45 ratio was prepared. The appropriate dose of insecticides was added to the suspension. Agrocoat was included to ensure the insecticide adhered well to the seeds. The seed was treated in the covered glass bottle until the intense red color of Agrocoat allowed the quality of the treatment to be checked. The amount of suspension required to treat a certain amount of seeds was determined from previous experiments in which the quantity of seed treated with a mixture of water and Agrocoat was measured. The seeds and treatment suspension were mixed in a closed system until all treated seeds had a uniform red color. After treatment, the seeds were dried on a kitchen towel and were ready for sowing.

Sowing was carried out in 500 mL plastic containers filled with sterile Gardol soil (BAUHAUS Services GmbH, Mannheim, Germany). Five seeds were sown in each container. In 2016, sowing took place on 27 May, and in 2017, on 13 May. Each treated and untreated seed was sown in five replicates in 2016 and four replicates in 2017.

Wireworm larvae were collected from maize fields in northwestern Croatia where heavy damage had previously been observed. Among the collected larvae, *Agriotes ustulatus* Shall larvae predominated. However, other *Agriotes* species were also present. Due to the difficulties in distinguishing larvae of other species belonging to the genus *Agriotes* (i.e., *Agriotes lineatus*, *Agriotes obscurus*, *Agriotes brevis*, and *Agriotes sputator*, which are the most abundant species in Croatia), we did not establish the exact species composition of the larvae used in the trial.

Five wireworm larvae were added to each seeded container on 11 June 2016 and 16 May 2017.

On 30 June 2016, and on 31 May 2017, all soil and plants from each container were emptied on a plastic coaster. All wireworm larvae were segregated and examined to establish the number of living larvae in each container. The efficacy of each treatment was calculated using the following formula by Abbott [75]:

(1) $$\%\;efficacy=100\times \frac{C-T}{C}$$

where

C = number of living larvae on the control;
T = number of living larvae on the treatment.

### 4.2. Sugar Beet Pests

Research was conducted in 2017 under laboratory conditions. The same insecticide products as for wireworms were used in the experiment: (i) Coragen 20 SC (DuPont), based on chlorantraniliprole from the anthranilic diamide group; (ii) Laser 240 KS (DowAgroSciences), based on spinosad from the spinosyn group; and (iii) NeemAzal (Trifolio-M GmbH), based on azadirachtin. The insecticide Actara 25 WG (Syngenta), based on thiamethoxam from the neonicotinoid group, was used as a standard. Each insecticide was applied in three different doses. For chlorantraniliprole, spinosad, and thiamethoxam, the applied doses were 0.6, 0.4, and 0.2 mg active ingredient per seed, and for azadirachtin, the doses were 12.9, 8.6, and 4.3 mg active ingredient per seed. An untreated control was also included.

The same seed treatment procedure as for maize was followed. Natural sugar beet seed, variety Pintea, was used in the trials. The amount of suspension required for treatment of 200 sugar beet seeds was 5 mL, as determined from previous experiments in which seeds were treated with a mixture of water and Agrocoat in a 55:45 ratio. A suspension of water and Agrocoat-TH Intensivrot 3855 in a 55:45 ratio was prepared, and the appropriate dose of insecticides was added to the suspension. Agrocoat was included to ensure the insecticide adhered well to the seeds. The intense red color of this product allowed the quality of the treatment to be checked. The seeds and treatment suspension were mixed in a closed system (plastic bag) and shaken until all treated seeds had a uniform red color. After treatment, the seeds were dried on a paper towel and were ready for sowing. The seeds were treated on 24 March 2017, and sowing was conducted every 10 days, on 28 March, 7 April, and 17 April 2017. The seeds were sown separately in 200 mL plastic containers. On each date, 60–70 containers with each treatment (insecticide dose and untreated) were sown to ensure the availability of plants for the trial.

CSALOMON^®^ TAL traps (modified pitfall traps) baited with aggregation pheromones for sugar beet weevil (Plant Protection Institute, CAR HAS, Budapest, Hungary) were set up on sugar beet fields in the area of Tovarnik, Croatia (45.169° N, 19.1522° E), on 10 April 2017. Three days later, on 13 April 2017, adults were collected from the traps and were transferred on the same day to the laboratory of the University of Zagreb Faculty of Agriculture.

The experimental setup for sugar beet weevil involved four replications, each containing one plastic container with a sugar beet plant grown from treated seed and 10 weevils released on one plant on 15 April 2017. At the time of the experimental setup, sugar beet plants had two pairs of leaves developed; they were at stage BBCH 12 [76]. Plastic containers were covered with transparent plastic containers of the same size to keep weevils on the plants. To ensure fresh air, the transparent containers had holes. Plastic containers were attached to each other with tape.

During the five days (120 h), the number of live and dead weevils was recorded, and if the plants were eaten, they were replaced with new undamaged plants. When replacement was necessary, plants from the same treatment and sowing cohort were used to maintain comparable exposure conditions. The efficacy of insecticide treatments against SBW was calculated at 96 and 120 h after exposure using the same formula as for wireworms.

Sugar beet flea beetles were collected on sugar beet fields in the area of Tovarnik on 2 May by using a mouth-operated suction-type aspirator. The same day, adults were transferred to the laboratory of the University of Zagreb Faculty of Agriculture.

The experimental setup for sugar beet flea beetles involved four replications, each containing one Petri dish with one leaf from sugar beet plants grown from treated or untreated seed and 10 flea beetles. On 3 May, the leaf was placed on wet filter paper on the bottom of the dish and beetles were released. A few hours before their release, the flea beetles were kept in the refrigerator at 4 °C to ensure successful handling and release.

During the four days (96 h), the number of live and dead flea beetles was recorded, and if the leaves were heavily damaged, they were replaced with new undamaged leaves. When leaves were replaced, fresh leaves from plants of the same treatment were used in order to preserve treatment consistency throughout the assay. The efficacy of insecticide treatments against SBFB was calculated at 48, 72 and 96 h after the exposure using the same formula as for wireworms.

### 4.3. Statistical Analysis

Data on the efficacy of insecticides against wireworms in both years, SBW, and SBFB were analyzed separately using two-way analysis of variance (ANOVA), with two factors, insecticide and dose; it was determined which of these factors and/or their interaction most affects efficiency. The mean values were ranked using Tukey’s test. Variance was tested with Levene’s test, and to achieve a normalized distribution, the 2017 efficiency data were transformed using a log (x + 1) (for wireworms), *√x + 0.5* and the *arcsin√x* transformation (for SBFB) before analysis of variance, as recommended when the mean value is positively correlated with variance [77]. These analyses were performed using the ARM 9 software package [78].

Statistical analysis of the survival of sugar beet weevil and sugar beet flea beetles was performed using the R software (version 4.0.3 (2020-10-10)) environment [79]. Survival probabilities were estimated using the Kaplan–Meier method, and differences between treatment groups were evaluated using the log-rank test via the survival package [80]. The Cox proportional hazards model was employed to assess the effect of treatments on mortality. All survival and cumulative mortality curves were visualized using the survminer [81] and ggplot2 [82] packages.

## 5. Conclusions

Based on the research results, we can conclude that only spinosad is a good candidate as a seed treatment insecticide that can ensure effective protection against wireworms. None of the investigated insecticides provided good protection against SBW. In contrast, SBFB could be successfully controlled with all the investigated seed treatment insecticides: chlorantraniliprole, spinosad, and azadirachtin. Despite the good efficacy against SBFB, the economic viability of the seed treatment formulation can be expected only if the treatment provides simultaneous protection against multiple pests. Therefore, future research should focus on investigating the possible efficacy of the studied insecticides on pests that also attack sugar beets and other field crops such as oilseed rape, maize, or sunflower, either in the soil or during early developmental phases. Possible target pests could include aphids, western corn rootworm, turnip sawfly, and others. The results indicate that replacing neonicotinoid seed treatments will likely require pest-specific or multi-component strategies rather than relying on a single alternative active substance.

## Figures and Tables

**Table 1:** Factorial analysis of data on the efficacy of insecticides on wireworms in trials conducted in 2016 and 2017.

Source of Variability		2016				2017		
	df	*p*	F	HSD	df	*p*	F	HSD
Total	36				36			
Rep	3	0.2930			3			
Active ingredient (A)	2	<0.01 **	257.020	6.45	2	<0.01 **	123.233	1.31
Dose (B)	2	<0.01 **	26.280	5.07	2	0.8575	0.154	2.51
AxB	4	<0.01 **	21.239	14.52	4	<0.01 **	6.465	6.02
Error	25				25			

df—degrees of freedom; *p*—probability value; HSD—honestly significant difference. ** Significant difference at 99 % probability.

**Table 2:** Efficacy of insecticide treatments against wireworms in trials in 2016 and 2017.

Insecticide Treatment	Dose (g of a.i/kg of Seed)	Efficacy (%)	
		2016	2017
Thiamethoxam	5	54.55 ± 10.46 b	13.25 ± 0.14 c
	3.5	36.37 ± 4.22 c	17.49 ± 0.22 bc
	2	40.91 ± 8.78 c	13.25 ± 0.14 c
Chlorantraniliprol	5	1.82 ± 1.66 e	2.18 ± 0.34 d
	3.5	15.97 ± 4.15 d	2.18 ± 0.34 d
	2	5.46 ± 0.74 ed	2.18 ± 0.34 d
Spinosad	5	72.73 ± 7.55 a	71.92 ± 0.05 a
	3.5	72.72 ± 7.55 a	49.56 ± 0.07 ab
	2	36.37 ± 7.55 c	28.69 ± 0.17 abc

Values marked with the same letter (a–e) do not differ significantly (*p* > 0.05; Tukey’s HSD. Equality of variances was tested with Levene’s test and reaches with equal variances (*p* > 0.05); mean descriptions for 2017 are reported in transformed data units and are not de-transformed (data were *log* *(x + 1)* transformed).

**Table 3:** Efficacy (%) of insecticides applied as seed treatment on sugar beet weevil (SBW) (±SD) in laboratory trial and results of the statistical analyses.

Active Ingredient	Dose (mg a.i./Seed)	96 h	120 h
thiamethoxam	0.6	25.82 ± 7.95 bc *	33.33 ± 10.10 ab
	0.4	32.43 ± 3.60 ab	36.11 ± 5.16 a
	0.2	16.22 ± 4.18 def	25.00 ± 9.47 bc
spinosad	0.6	7.43 ± 2.20 g	11.11 ± 2.99 de
	0.4	18.92 ± 5.79 cde	19.19 ± 6.13 cd
	0.2	24.32 ± 0.00 c	25.00 ± 5.56 bc
chlorantraniliprole	0.6	32.43 ± 3.60 ab	33.33 ± 3.63 ab
	0.4	37.84 ± 5.96 a	39.14 ± 7.73 a
	0.2	10.14 ± 4.52 fg	8.33 ± 2.72 e
azadirachtin	0.6	13.51 ± 2.30 efg	19.44 ± 5.63 cd
	0.4	21.62 ± 3.60 cd	25.00 ± 2.29 bc
	0.2	21.62 ± 8.05 cd	30.56 ± 10.64 ab
^1^ HSD*_p_*_=5%_		7.069	9.60

^1^ HSD within columns was determined by Tukey’s test (*p* = 0.05). Values marked with different lowercase letters are significantly different within columns. * significant difference at 95% probability.

**Table 4:** Pair-wise comparisons (*p*-values) of survival curves for sugar beet weevils (SBW) 120 h after exposure to different insecticides.

**Thiamethoxam**	**Probability (*p*)**		
	**Untreated**	**Thiamethoxam 0.2**	**Thiamethoxam 0.4**
Thiamethoxam 0.2	<0.05 *	-	-
Thiamethoxam 0.4	<0.05	0.4727	-
Thiamethoxam 0.6	<0.05	0.4796	0.7524
**Chlorantraniliprole**	**Probability (*p*)**		
	**Untreated**	**Chlorantraniliprole 0.2**	**Chlorantraniliprole 0.4**
Chlorantraniliprole 0.2	0.5821	-	-
Chlorantraniliprole 0.4	<0.01 **	<0.05 *	-
Chlorantraniliprole 0.6	<0.01 **	<0.01 **	0.7647
**Spinosad**	**Probability (*p*)**		
	**Untreated**	**Spinosad 0.2**	**Spinosad 0.6**
Spinosad 0.2	0.081	-	-
Spinosad 0.4	0.138	0.590	-
Spinosad 0.6	0.334	0.334	0.475
**Azadirachtin**	**Probability (*p*)**		
	**Untreated**	**Azadirachtin 4.3**	**Azadirachtin 8.6**
Azadirachtin 4.3	<0.05 *	-	-
Azadirachtin 8.6	<0.05 *	0.627	-
Azadirachtin 12.9	0.092	0.497	0.662

* Significant difference at 95% probability. ** Significant difference at 99% probability.

**Table 5:** Efficacy (%) of insecticides applied as seed treatment on sugar beet flea beetle (SBFB) (±SD) in laboratory trial and results of the statistical analyses.

Active Ingredient	Dose (mg a.i./Seed)	Efficacy (%) ± SD		
		48 h *	72 h	96 h **
thiamethoxam	0.6	18.78 ± 0.16 cd	39.39 ± 3.45 d	78.84 ± 6.41 de
	0.4	32.07 ± 0.33 a	57.58 ± 5.58 b	84.61 ± 5.95 cd
	0.2	20.64 ± 0.35 c	39.99 ± 6.85 d	64.94 ± 3.71 e
spinosad	0.6	11.41 ± 0.22 f	60.63 ± 9.17 ab	69.98 ± 0.41 de
	0.4	2.14 ± 0.09 i	60.63 ± 6.79 ab	99.43 ± 9.36 ab
	0.2	4.30 ± 0.08 h	24.26 ± 4.80 e	97.36 ± 10.81 ab
chlorantraniliprole	0.6	14.25 ± 0.26 e	45.45 ± 5.17 cd	71.00 ± 5.18 de
	0.4	4.91 ± 0.36 h	39.40 ± 1.72 d	63.4 ± 3.51 e
	0.2	9.29 ± 0.22 g	48.50 ± 1.80 c	94.11 ± 9.36 bc
azadirachtin	12.9	26.28 ± 0.15 b	66.70 ± 5.41 a	100.00 ± 0.00 a
	8.6	17.39 ± 0.28 d	62.64 ± 5.11 ab	99.33 ± 9.36 ab
	4.3	0.00 ± 0.00 j	42.43 ± 7.87 cd	77.28 ± 6.76 de
^1^ LSD*_p_*_=5%_		1089	7201	2874

^1^ LSD within columns was determined by Tukey’s HSD (*p* < 0.05). Values marked with different lowercase letters are significantly different within columns. * To achieve a uniform distribution, data were transformed using *√x + 0.5* before analysis of variance. ** To achieve a uniform distribution, data were transformed using the *arcsin√x* transformation before analysis of variance.

**Table 6:** Pair-wise comparisons (*p*-values) of survival curves for sugar beet flea beetles (SBFB) 96 h after exposure to different insecticides.

**Thiamethoxam**	**Probability (*p*)**		
	**Untreated**	**Thiamethoxam 0.2**	**Thiamethoxam 0.4**
Thiamethoxam 0.2	<0.01 **	-	-
Thiamethoxam 0.4	<0.01 **	0.11	-
Thiamethoxam 0.6	<0.01 **	0.51	0.26
**Chlorantraniliprole**	**Probability (*p*)**		
	**Untreated**	**Chlorantraniliprole 0.2**	**Chlorantraniliprole 0.4**
Chlorantraniliprole 0.2	<0.01 **	-	-
Chlorantraniliprole 0.4	<0.01 **	0.12343	-
Chlorantraniliprole 0.6	<0.01 **	0.43301	0.43301
**Spinosad**	**Probability (*p*)**		
	**Untreated**	**Spinosad 0.2**	**Spinosad 0.6**
Spinosad 0.2	<0.01 **	-	-
Spinosad 0.4	<0.01 **	0.052	-
Spinosad 0.6	<0.01 **	0.592	0.497
**Azadirachtin**	**Probability (*p*)**		
	**Untreated**	**Azadirachtin 4.3**	**Azadirachtin 8.6**
Azadirachtin 4.3	<0.01 **	-	-
Azadirachtin 8.6	<0.01 **	<0.01 **	-
Azadirachtin 12.9	<0.01 **	<0.01 **	0.2392

** Significant difference at 99% probability.

**Table 7:** Overview of products, active ingredients, and doses used in the maize seed treatment trial.

Insecticide/Producer	Active Ingredient	Dose	Year	
			2016	2017
Actara 25 WG/Syngenta	thiamethoxam	5 g a.i./1 kg seed	+	+
		3.5 g a.i./1 kg seed	+	+
		2 g a.i./1 kg seed	+	+
Coragen 20 SC/DuPont	chlorantraniliprole	5 g a.i./1 kg seed	+	+
		3.5 g a.i./1 kg seed	+	+
		2 g a.i./1 kg seed	+	+
Laser 240 SC/Corteva	spinosad	5 g a.i./1 kg seed	+	+
		3.5 g a.i./1 kg seed	+	+
		2 g a.i./1 kg seed	+	+
NeemAzal/Trifolio-M GmbH	azadirachtin	19.38 mg a.i./seed	−	+
		12.92 mg a.i./seed	−	+
		6.46 mg a.i./seed	−	+

## Data Availability

The original contributions presented in this study are included in the article. Further inquiries can be directed to the corresponding authors.
